# Thermodynamically Stable Cationic Dimers in Carboxyl-Functionalized Ionic Liquids: The Paradoxical Case of “Anti-Electrostatic” Hydrogen Bonding

**DOI:** 10.3390/molecules27020366

**Published:** 2022-01-07

**Authors:** Loai Al-Sheakh, Sebastian Fritsch, Andreas Appelhagen, Alexander Villinger, Ralf Ludwig

**Affiliations:** 1 Institut für Chemie, Abteilung für Physikalische Chemie, Universität Rostock, Dr.-Lorenz-Weg 2, 18059 Rostock, Germany; loai.sheakh2@uni-rostock.de (L.A.-S.); sebastian.fritsch@uni-rostock.de (S.F.); andreas.appelhagen@uni-rostock.de (A.A.); 2 Institut für Chemie, Abteilung für Anorganische Chemie, Universität Rostock, Albert-Einstein-Str. 3a, 18059 Rostock, Germany; alexander.villinger@uni-rostock.de; 3 Department LL&M, University of Rostock, Albert-Einstein−Str. 25, 18059 Rostock, Germany; 4 Leibniz−Institut für Katalyse an der Universität Rostock e.V., Albert-Einstein−Str. 29a, 18059 Rostock, Germany

**Keywords:** “anti-electrostatic” hydrogen bonds, carboxyl-functionalized ionic liquids, cationic dimers, NBO analysis, QCE theory

## Abstract

We show that carboxyl-functionalized ionic liquids (ILs) form doubly hydrogen-bonded cationic dimers (c^+^=c^+^) despite the repulsive forces between ions of like charge and competing hydrogen bonds between cation and anion (c^+^–a^−^). This structural motif as known for formic acid, the archetype of double hydrogen bridges, is present in the solid state of the IL 1−(carboxymethyl)pyridinium bis(trifluoromethylsulfonyl)imide [HOOC−CH_2_−py][NTf_2_]. By means of quantum chemical calculations, we explored different hydrogen-bonded isomers of neutral (HOOC–(CH_2_)*_n_*–py^+^)_2_(NTf_2_^−^)_2_, single-charged (HOOC–(CH_2_)*_n_*–py^+^)_2_(NTf_2_^−^), and double-charged (HOOC– (CH_2_)*_n_*−py^+^)_2_ complexes for demonstrating the paradoxical case of “anti-electrostatic” hydrogen bonding (AEHB) between ions of like charge. For the pure doubly hydrogen-bonded cationic dimers (HOOC– (CH_2_)*_n_*−py^+^)_2_, we report robust kinetic stability for *n* = 1–4. At *n* = 5, hydrogen bonding and dispersion fully compensate for the repulsive Coulomb forces between the cations, allowing for the quantification of the two equivalent hydrogen bonds and dispersion interaction in the order of 58.5 and 11 kJmol^−1^, respectively. For *n* = 6–8, we calculated negative free energies for temperatures below 47, 80, and 114 K, respectively. Quantum cluster equilibrium (QCE) theory predicts the equilibria between cationic monomers and dimers by considering the intermolecular interaction between the species, leading to thermodynamic stability at even higher temperatures. We rationalize the H-bond characteristics of the cationic dimers by the natural bond orbital (NBO) approach, emphasizing the strong correlation between NBO-based and spectroscopic descriptors, such as NMR chemical shifts and vibrational frequencies.

## 1. Introduction

The common wisdom that “unlike charges attract, like charges repel” has been recently challenged by Weinhold and Klein [1]. Employing quantum chemistry techniques, they characterized a surprising new class of hydrogen-bonded complexes between ions of like charge. Based on ab initio and density functional theory (DFT) calculations, they claimed that doubly charged complexes [A−HB]^2±^ are manifestations of “anti-electrostatic” hydrogen bonds (AEHB), wherein the short-range donor−acceptor covalency forces overcome the powerful long-range electrostatic opposition to be expected between ions of like charge [2,3,4,5,6,7]. Potential energy curves for the ion−ion interactions showed shallow local minima, indicating hydrogen bonding between the like-charged ions and kinetic stabilization. Searching for robustly bonded high-order AEHB aggregates, Weinhold investigated doubly hydrogen-bonded hydrogen bisulfate dimers (HSO_4_^−^)_2_ and dihydrogen biphosphate dimers (H_2_PO_4_^−^)_2_ [8]. However, even the extremely strong hydrogen bonds in both anionic dimers still exhibited positive energies of Δ*E* = 164.8 kJmol^−1^ and Δ*E* = 135.8 kJmol^−1^ above the asymptotic limit of infinitely separated ions (Δ*E* = 0). Nevertheless, these dimers are remarkably metastable with robust binding wells ranging between 13 and 36 kJmol^−1^, exceeding by ca. 100-fold the corresponding value (0.2 kJmol^−1^) of the analogous (F^−^···HCO_3_^−^) complex [1,2,3]. The stability of the bisulfate and biphosphate anion dimers was discussed before by Mata et al., but attributed to “electrophilic−nucleophilic interactions” rather than charge transfer as an important factor in H-bonding [9,10,11]. Although these anionic dimers show robust binding wells that are stabilized by broad potential barriers opposing “Coulomb explosion” to separated ions, these (a^−^=a^−^) complexes are far away from thermodynamic stability and not accessible by experiment at any pressure and temperature. Recently, we suggested to consider anionic dimers (HOOC−(CH_2_)*_n_*−COO^−^)_2_ derived from singly deprotonated dicarboxylic acids, such as adipic acid (*n* = 4), which is a precursor used in the production of nylon [12]. These calculated dimers (a^−^=a^−^) show thermodynamic stability for *n* = 6,7, but ionic liquids including carboxyl-functionalized anions showing this structural feature could not be synthesized so far.

Inspired by Weinhold’s idea of existing “antielectrostatic” hydrogen bonds (AEHB), we started to seek experimental evidence of this elusive concept. Until then, like-charge attraction in “real systems” had been reported mainly for large-scale structures, assemblies, or stabilizing frameworks [13,14,15,16,17,18,19,20,21]. Overall attractive interaction between the “likes” in liquids and solutions required screening effects caused by neighboring counterions, polar molecules, or generally favorable dielectric environments [22,23,24,25,26,27,28,29,30,31,32,33,34,35]. Our attempts aimed not only to detect but to control like-charge attraction by varying molecular parameters. The first cationic clusters we observed accidentally in the infrared (IR) spectra of the ionic liquid 1,1,1−trimethyl−1−(2−hydroxyethyl) ammonium bis(trifluoromethylsulfonyl)imide. The redshift of the OH vibrational modes in (c^+^–c^+^) cationic dimers were comparable to those known for ethanol dimers [22]. Switching to the IL 1−(2−hydroxyethyl)−3−methylimidazolium tetrafluoroborate, we detected well-distinguished vibrational bands representing hydrogen bonding between cation and anion (c^+^–a^−^) and between cations (c^+^–c^+^), respectively. Using these polarizable hydroxyl-functionalized imidazolium cations, the populations strongly shifted towards (c^+^–c^+^) cluster formation in particular at low temperatures [23,24,25]. Considering all favorable properties of possible cations and anions for enhanced (c^+^–c^+^) hydrogen bonding interaction, namely, weakly interacting anions, polarizable cations, and sufficiently long hydroxyalkyl chains, we observed reasonable amounts of cationic clusters already at room temperature for the IL 1−(4−hydroxybutyl-pyridinium) bis(trifluoromethylsulfonyl)imide [HO−(CH_2_)_4_Py][NTf_2_] [26,27,28,29,30,31,32,33,34,35]. For the latter IL, we characterized the hydrogen bonds between ions of opposite charge (c^+^−a^−^) and ions of like charge (c^+^–c^+^) by means of neutron scattering isotopic substitution (NDIS) experiments [28]. We showed that the (c^+^−c^+^) H-bond distances *R*(H···O) and *R*(O···O) were about 10 pm shorter than those of the charge-assisted (c^+^−a^−^) H-bonds in accordance with stronger redshifted vibrational bands observed in the IR spectra and smaller deuteron quadrupole coupling constants detected in NMR solid-state experiments [29,30,31]. Obviously, hydrogen bonds between ions of like charge can be stronger than those between ions of opposite charge despite the repulsion in the first interaction and the attractive Coulomb interaction in the latter case. Recently, we showed by means of molecular dynamics (MD) simulations that the hydrogen bonding lifetimes of the (c^+^–c^+^) H-bonds can be longer than those of the (c^+^–a^−^) H-bonds for alkyl chain length *n* = 4–5, reflecting the different interaction strengths in both types of hydrogen bonds, as indicated by IR and NMR spectroscopy [32,33,34,35]. However, the populations derived from neutron diffraction data showed that only less than 20% of all hydrogen bonds refer to (c^+^–c^+^) bound clusters at room temperature. Moreover, we only had indirect information about the size of the (c^+^–c^+^) cluster species and their specific binding motifs. This could be clarified in a collaborative work with Mark Johnson’s group at Yale University. They developed a cryogenic ion vibrational predissociation (CIVP) strategy, using supersonic jet ion sources that readily access low temperatures, and yielding structures for many species, such as hydrated protons, hydrated electrons, hydroxide ions, but also charged clusters of ionic liquids [36,37,38,39]. For hydroxyl-functionalized ILs, they were also able to detect positively charged clusters from ionic liquids exhibiting hydrogen bonding between ions of like charge [40,41,42,43]. Menges et al. observed isolated ion clusters in the gas phase consisting of two 1–(2–hydroxyethyl)–3–methylimidazolium cations (HEMIm^+^) and one bis(trifluoromethylsulfonyl)imide anion (NTf_2_^−^) by means of CIVP spectroscopy [40]. Supported by double resonance techniques, they showed that the two cations are linked through hydrogen bonding, while one OH is attached to an oxygen of the anion, resulting in positively charged (HEMIm^+^)_2_(NTf_2_^−^)_1_ clusters of type (c^+^–c^+^–a^−^). In larger complexes (HEMIm^+^)_3_(NTf_2_^−^)_2_, even contact between three cations was detected in (c^+^–c^+^–c^+^–a^−^)(a^−^) isomers [41]. However, the counterions still play a crucial role for the stability of the singly charged (2,1) and (3,2) complexes, as shown by accompanying quantum chemical calculations [42,43,44,45,46,47,48].

It became obvious that for studying like-charge attraction in singly charged and even doubly charged cationic clusters in more detail, stronger H-bonding between the ions of like charge is required. This brought us to the idea of using carboxyl-functionalized cations for providing two strong hydrogen bonds between the cations, strongly enhancing the attractive interaction and overcoming the repulsive Coulomb forces. This very productive cation–cation motif with two carboxylate groups in self-complementary C_2_-symmetric interaction geometry gives antiparallel-bridging H-bonds that benefit from the powerful cooperativity of coupled donor–acceptor interactions. We just showed for the pharmaceutical ibuprofen, which exhibits the same structural H-bond motif, that each of the two H-bonds is about 10% stronger than it would be alone [49]. In this work, we try to realize the formation of this cation–cation HB motif in ionic liquids comprising carboxyl-functionalized cations and weakly interacting anions [50,51,52]. After showing that this favorable HB motif is already present in the solid state of the ionic liquid 1-(carboxymethyl)pyridinium bis(trifluoromethylsulfonyl)imide [HOOC–CH_2_–py][NTf_2_], we used quantum chemistry for studying the strength of this HB motif in single positive (HOOC–CH_2_–py^+^)_2_NTf_2_^−^ and double positive (HOOC–CH_2_–py^+^)_2_ complexes, wherein in principle the formation of other HB configurations is possible. The HB strength in the pure cationic dimers (HOOC–(CH_2_)*_n_*–py^+^)_2_ with *n* = 1–8 is then investigated for increasing alkyl chain length, reducing the Coulomb repulsion between the ions of like charge. The ultimate goal here is to identify the first thermodynamically stable cationic dimers, wherein short-ranged hydrogen bonds overcome long-ranged repulsive Coulomb interaction, examining the electrostatic-based concepts of hydrogen bonding. In particular, we consider thermodynamically stable cationic dimers (HOOC–(CH_2_)*_n_*–py^+^)_2_, which could be obtained from ILs [HOOC–(CH_2_)*_n_*–py][NTf_2_] that are detectable by CIVP spectroscopy at a temperature around 30 K. In this respect, the quantum cluster equilibrium (QCE) model provides equilibria of monomeric and dimeric complexes including explicitly intermolecular interaction [53,54,55]. Finally, we rationalize the H-bond characteristics of the dimers by the natural bond orbital (NBO) approach, emphasizing the strong correlation between NBO-based and spectroscopic descriptors, such as NMR chemical shifts and vibrational frequencies [56,57,58,59,60].

## 2. Materials and Methods

### 2.1. Synthesis and Characterization

The ionic liquid 1-(carboxymethyl)pyridinium bis(trifluoromethylsulfonyl)imide [HOOC–CH_2_–py][NTf_2_] was synthesized as reported in the literature [61]. A metathesis reaction was used to exchange the chloride anions for a bis(trifluoromethylsulfonyl)imide anion, starting from the commercially available 1-(carboxymethyl)pyridinium hydrochloride (Sigma-Aldrich, Taufkirchen, Germany). The ILs have a melting point of 32 °C.

An X-ray quality crystal of the ionic liquid 1-(carboxymethyl)pyridinium bis(trifluoromethylsulfonyl)imide [HOOC–CH_2_–py][NTf_2_] was prepared in Fomblin YR-1800 perfluoropolyether (Alfa Aesar, Kandel, Germany) at ambient temperature. The sample was cooled to 123(2) K during measurement. Data were collected on a Bruker KAPPA APEX II diffractometer using Mo Kα radiation (λ = 0.71073 Å). The structures were solved by direct methods (SHELXS-2013) and refined by full-matrix least squares procedures (SHELXL-2013) [62,63]. Semiempirical absorption corrections were applied (SADABS) [64]. All non-hydrogen atoms were refined anisotropically, and hydrogen atoms were included in the refinement at calculated positions using a riding model.

### 2.2. Computational Methods

**Scheme 1:**
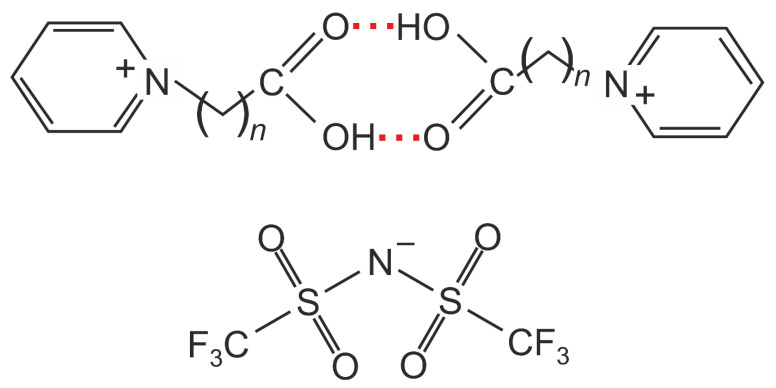
Structural motif of doubly hydrogen-bonded cationic dimers [HOOC–(CH_2_)*_n_*–py^+^]_2_ as calculated for different numbers of methylene groups with *n* = 1–8. Below, we show the weakly interacting anion bis(trifluoromethylsulfonyl)imide [NTf_2_^−^] as considered in the calculations of neutral and charged clusters and present in the ionic liquid [HOOC–CH_2_–py][NTf_2_].

We calculated clusters’ different hydrogen-bonded isomers of neutral (HOOC–(CH_2_)*_n_*–py^+^)_2_(NTf_2_^−^)_2_, single-charged (HOOC–(CH_2_)*_n_*–py^+^)_2_(NTf_2_^−^), and double-charged (HOOC–(CH_2_)*_n_*–py^+^)_2_ complexes for demonstrating the paradoxical case of “anti-electrostatic” hydrogen bonding (AEHB) between ions of like charge (see Scheme 1).

For the neutral and cationic clusters, we employed B3LYP/6-31+G* and B3LYP-D3/6-31+G* calculations performed with the Gaussian 09 program [65]. For calculating the clusters at the same level of theory, we used the well-balanced 6-31+G* Pople basis set. Including polarization and diffuse functions, this basis set is suitable for reasonably calculating hydrogen-bonded clusters of like-charged ions. We used the relatively small 6-31+G* basis set for calculating all clusters at the same level of theory as well as for better comparison with earlier studies of molecular and ionic clusters [44,45,46,47,48]. We demonstrated that the salient properties of these clusters can be robustly calculated with both smaller and larger basis sets as long as Grimme’s D3 dispersion correction is considered [66,67,68]. All clusters were fully optimized, followed by frequency calculations. The obtained vibrational frequencies were all positive, showing that we calculated at least local minimum structures. The NMR proton chemical shifts δ^1^H were calculated at the same level of theory.

The pure cationic dimers were additionally calculated at the dispersion-corrected B3LYP–D3/def2–TZVP and the ab initio Møller Plesset MP2/6–31+G* levels of theory for demonstrating the relevance of method and basis set uncertainties for the binding energies [69,70]. Overall, we can state that the influence of used methods and basis sets is not larger than the effect of adding or removing one methylene group (±3 kJmol^−1^).

We also want to point out that the counterpoise (CP) correction of Boys and Bernardi has been questioned recently [71,72,73]. Intrinsic CP artifacts for ion pair clusters add to fundamental concerns that the CP procedure is generally unreliable for correcting the basis set superposition error (BSSE) [72]. Nevertheless, we applied a CP correction to the calculated dimers for demonstrating the minor role of the BSSE correction for the resulting structures and energies if well-balanced basis sets are used.

### 2.3. Strategy of This Study and Investigated Complexes

**Scheme 2:**
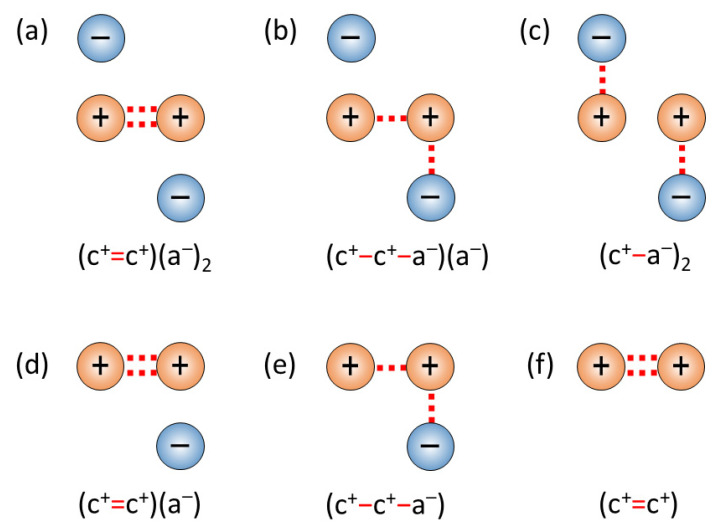
Illustration of competing interactions at play in ionic liquids comprising carboxyl-functionalized cations in the neutral complexes (**a**–**c**), the single positively charged complexes (**d**,**e**), and the double positively charged complexes (**f**).

We explore complexes supposed to be present in the solid, liquid, and gaseous phases of the ionic liquid 1–(*n*–carboxyalkyl)pyridinium bis(trifluoromethylsulfonyl)imide [HOOC–(CH_2_)*_n_*–py][NTf_2_] (see Scheme 1). The neutral and the singly charged complexes are studied for *n* = 1, whereas the doubly charged pure cationic complexes are calculated for *n* = 1–8. Possible isomers of the differently charged complexes are illustrated in Scheme 2.

For the neutral complex (HOOC–CH_2_–py^+^)_2_(NTf_2_^−^])_2_, three types of hydrogen bonding motifs might be formed: (a) Two cations HOOC–CH_2_–py^+^ are doubly hydrogen-bonded with each other, and two anions are attached to the anions without specific interaction, denoted as complex (c^+^=c^+^)(a^−^)^2^. (b) In a (c^+^–c^+^–a^−^)(a^−^) complex, the OH group on one cation HOOC–CH_2_–py^+^ binds to the carboxyl group CO on the other, while the OH group of the other attaches an oxygen atom of the anion. (c) Both cations form single hydrogen bonds to one of the anions without any hydrogen bond formed among them, denoted as (c^+^–a^−^)^2^.

For the singly charged cationic complexes (HOOC–CH_2_–py^+^)_2_NTf_2_^−^, wherein one counterion is removed from the neutral complex, two types of hydrogen bonding motifs are possible: (d) Two cations HOOC–CH_2_–py^+^ are still doubly hydrogen-bonded, but only one anion is present, leading to the cationic complex denoted as (c^+^=c^+^)(a^−^). (e) In the other possible complex (c^+^–c^+^–a^−^), the OH group on one cation HOOC–CH_2_–py^+^ binds to the carboxyl group CO on the other, while the OH group of the other attaches an oxygen atom of the anion, similar to the neutral complex after removing one counterion.

The doubly charged cationic complexes (HOOC–CH_2_–py^+^)_2_, where all the counterions are released, are denoted as (c^+^=c^+^). Singly bound cationic complexes (c^+^–c^+^) can be ruled out because there, one donor and one acceptor function are free and result in a significantly less attractive interaction not capable of competing with the strong repulsive Coulomb forces between ions of like charge.

## 3. Results and Discussion

### 3.1. The Crystal Structure of the Ionic Liquid 1–(Carboxymethyl)Pyridinium Bis(Trifluoromethylsulfonyl)Imide [HOOC–CH_2_–py][NTf_2_]

**Figure 1:**
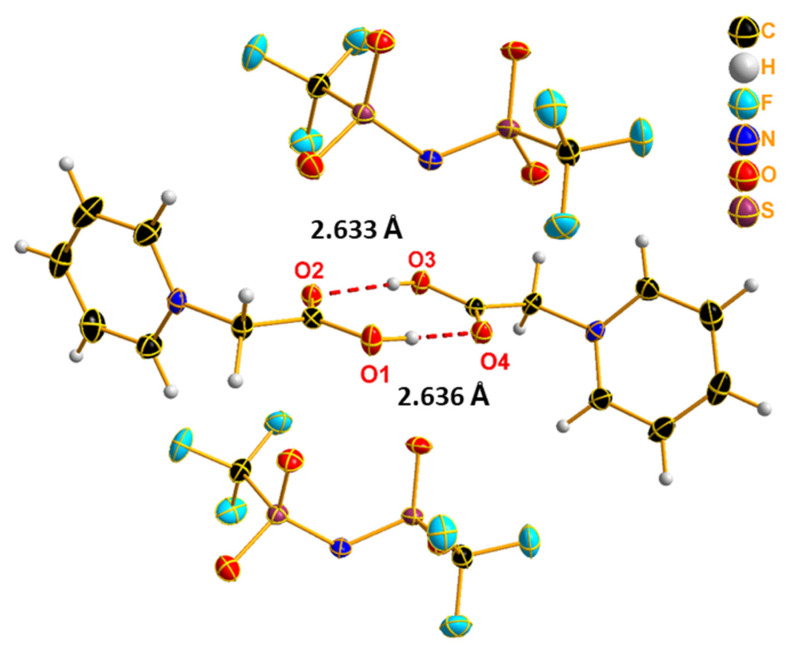
Solid-state structure of the ionic liquid 1–(carboxymethyl)pyridinium bis(trifluoromethylsulfonyl)imide [HOOC–CH_2_–py][NTf_2_]. The X–ray structure shows the favorable double H-bond motif formed between the carboxyl-functionalized cations, whereas the weakly interacting anions are not involved in hydrogen bonding. In the ORTEP representation, the atoms are represented by ellipsoids at 50% probability levels. The hydrogen bonds are denoted by a dashed line.

We synthesized the ionic liquid 1–(carboxymethyl)pyridinium bis(trifluoromethylsulfonyl)imide [HOOC–CH_2_–py][NTf_2_] following a literature method by Nockemann et al. [61]. The carboxyl-functionalized IL has a melting point of about 32 °C and can be easily crystallized. The X-ray structure shows the characteristic double hydrogen bonding motif between the two carboxyl groups of the cations, whereas the two weakly interacting bis(trifluoromethylsulfonyl)imide anions are attached but not involved in hydrogen bonding with any of the cations, as shown in Figure 1 (see Supplementary Materials). This structural motif as known for formic acid, the archetype of double hydrogen bridges, is characterized by two strong hydrogen bonds indicated by two short and almost similar *R*(O···O) distances between the hydroxyl oxygen and the carbonyl oxygen of about *R*(O2···O3) = 2.633 Å and *R*(O1···O4) = 2.636 Å, respectively (see Figure 1). Obviously, the two strong hydrogen bonds overcome the repulsive Coulomb forces between the carboxyl-functionalized cations and successfully compete with hydrogen bonding between cation and anion, although the latter is enhanced by attractive Coulomb interaction. For simple hydroxyl-functionalized ionic liquids, we showed earlier that strong cationic cluster formation prevents crystallization, resulting in supercooled and finally glassy systems, whereas weak cationic cluster formation completely disappears in the solid state and only hydrogen bonding between cation and anion remains [26,31]. This is not the case in the solid IL [HOOC–CH_2_–py][NTf_2_], where two strong hydrogen bonds in the motif (c^+^=c^+^) are obviously stronger than the possible single cation–cation (c^+^–c^+^) and cation–anion (c^+^–a^−^) hydrogen bonds between the molecular ions. We plan to investigate by IR and NMR spectroscopy whether the (c^+^=c^+^) HB bond motif survives the phase transition from the solid to the liquid phase and whether it remains stable with increasing temperature. The synthesis of ILs [HOOC–(CH_2_)*_n_*–py][NTf_2_] with *n* > 1 for decreasing Coulomb repulsion due to methylene group spacers and increasing the HB strength at the same time is in progress in our lab.

The occurrence of strong like-charge attraction already in the solid state raises the question of whether the characteristic H-bond motif will survive if the counterions are removed successively, leading to singly charged cationic complexes (HOOC–CH_2_–py^+^)_2_(NTf_2_^−^) and finally pure cationic complexes (HOOC–(CH_2_)*_n_*–py^+^)_2_.

### 3.2. The Structural Motif of Neutral (HOOC–(CH_2_)_n_–py^+^)_2_(NTf_2_^−^)_2_ Complexes

**Figure 2:**
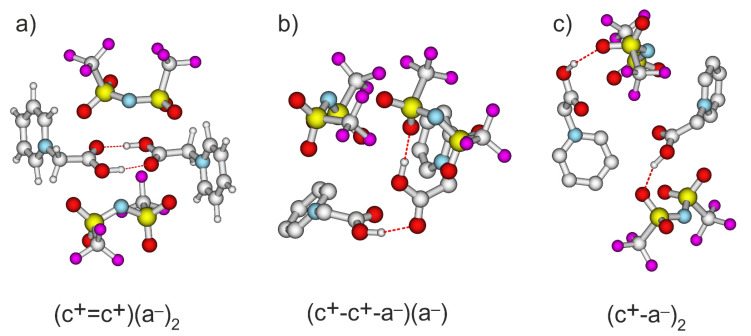
Structures of neutral complexes (HOOC–CH_2_–py^+^)_2_(NTf_2_^−^)_2_ as calculated at the B3LYP–D3/6–31+G* level of theory. We considered three types of hydrogen bonding motifs (from left to right). (**a**) Two cations HOOC–CH_2_–py^+^ are doubly hydrogen-bonded with each other, and two anions are attached to the cations without specific interaction, denoted as complex (c^+^=c^+^)(a^−^)^2^. (**b**) In a (c^+^–c^+^–a^−^)(a^−^) complex, the OH group on one cation HOOC–CH_2_–py^+^ binds to the carboxyl group CO on the other, while the OH group of the other attaches an oxygen atom of the anion. (**c**) Both cations form single hydrogen bonds to one of the anions without any hydrogen bond formed among them, denoted as (c^+^–a^−^)^2^.

For showing the reliability of the chosen quantum chemical level B3LYP–D3/6–31+G*, we compared the geometry of a calculated neutral dimer (HOOC–CH_2_–py)_2_(NTf_2_)_2_ of type (c^+^=c^+^)(a^−^)^2^ with the solid-state structure of the IL [HOOC–CH_2_–py][NTf_2_], as shown in Figure 2 [61,62,63,64]. Calculated *R*(O···O) distances of about 2.620 and 2.631 Å were in almost perfect agreement with the measured solid-state value of about 2.633 and 2.636 Å, respectively. The corresponding hydrogen bonding distances *R*(H···O) were calculated to be 1.689 Å with the related intramolecular bond length *R*(OH) = 1.008 Å. Thus, the OH bond length is comparable to that in ice, indicating strong hydrogen bonding.

Overall, the hydrogen bond distances *R*(O···O) in the present IL are significantly smaller than those observed in X-ray structures of crystalline carbonic and acetic acid structures [74,75]. However, the solid-state structure of the carboxyl-functionalized molecular acids reveal linear chain hydrogen bonds instead of the two strong hydrogen bonds. What we can state here is that hydrogen bonds between ions of like charge in the (c^+^=c^+^) dimer are stronger than the single hydrogen bonds in the molecular system despite the strong repulsive forces [46]. Besides the complex (c^+^=c^+^)(a^−^)^2^, resembling the structural motif observed in the crystal structure of the IL [HOOC–CH_2_–py][NTf_2_], we calculated other possible neutral complexes, such as (c^+^–c^+^–a^−^)(a^−^) and (c^+^–a^−^)^2^, as presented in Figure 2. The cluster (c^+^=c^+^)(a^−^)^2^ is 37.1 kJmol^−1^ lower in energy than the complex (c^+^–c^+^–a^−^)(a^−^), and the latter another 23.8 kJmol^−1^ lower than the complex (c^+^–a^−^)^2^, which includes two H-bonded ion pairs. This result strongly supports the stability of the double-hydrogen bonding motif as observed in the X–ray patterns of the solid IL.

### 3.3. The Structural Motif of Singly Charged (HOOC–CH_2_–py^+^)_2_(NTf_2_^−^) Complexes

**Figure 3:**
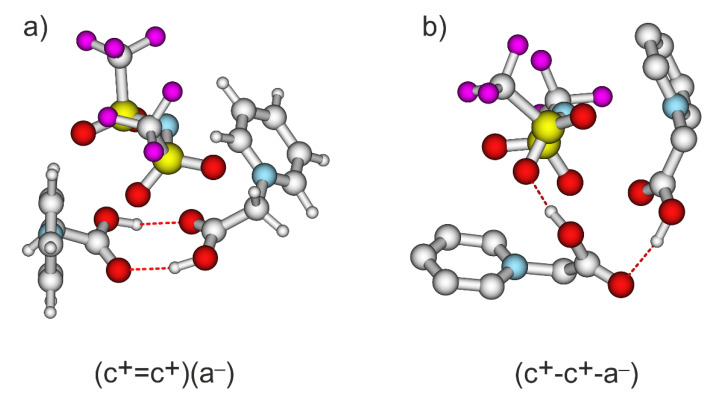
Structures of the positively charged complexes (HOOC–CH_2_–py^+^)_2_(NTf_2_^−^) as calculated at the B3LYP–D3/6–31+G* level of theory (from left to right). Two main H-bonded configurations are calculated: (**a**) complex (c^+^=c^+^)(a^−^), exhibiting the coupled donor–acceptor interactions, and (**b**) complex (c^+^–c^+^–a^−^), wherein the OH group on one cation HOOC–CH_2_–py^+^ binds to the carboxyl group CO on the other, while the OH group of the other attaches an oxygen atom of the anion.

We now address the question of whether the anti-parallel-bridging H-bonds between the two carboxylic groups remain if one counterion from the overall neutral complex (c^+^)^2^(a^−^)^2^ is removed. Two main H-bonded configurations are possible: complex (c^+^=c^+^)(a^−^), still including the coupled donor–acceptor interactions, and complex (c^+^–c^+^–a^−^), wherein the OH group on one cation HOOC–CH_2_–py^+^ binds to the carboxyl group CO on the other, while the OH group of the other attaches to an oxygen atom of the anion (see Figure 3). The latter type of H-bonded clusters was observed by Menges et al., applying CIVP spectroscopy at 30 K. They detected (2,1) complexes comprising two 1–(2–hydroxyethyl)–3–methylimidazolium cations and one bis(trifluoromethylsulfonyl)imide anion [40]. Combined with double resonance techniques, they showed that in some clusters the two cations are linked through hydrogen bonding, while one OH is attached to an oxygen of the anion, resulting in (c^+^–c^+^–a^−^) complexes. In larger complexes (HEMIm^+^)_3_(NTf_2_^−^)_2_, even contact between three cations was detected in (c^+^–c^+^–c^+^–a^−^)(a^−^) isomers [41]. We confirmed by quantum chemical calculations that the presence of counterions is crucial for the stability of the singly charged (2,1) and (3,2) complexes [44,45,46,47,48]. For pure cationic dimers (HEPy^+^)_2_, we calculated robust kinetic stability but were still far away from thermodynamic stability [44,46]. Even substantial lengthening of the hydroxyalkyl chains for reducing repulsive interaction and enhancing hydrogen bonding results in only energetically but not thermodynamically stable dimers. However, for the singly charged (HOOC–CH_2_–py^+^)_2_(NTf_2_^−^) complexes, we find that the structural motif (c^+^=c^+^)(a^−^) remains stable after removing one counterion. It is about 29.5 kJmol^−1^ lower in energy than the (c^+^–c^+^–a^−^) complex. Thus, the cooperative double H-bond is stronger than the two single H-bonds between the cations and the cation and the anion.

### 3.4. The Structural Motif of Double-Charged Cationic Dimer (HOOC–CH_2_–py^+^)_2_

**Figure 4:**
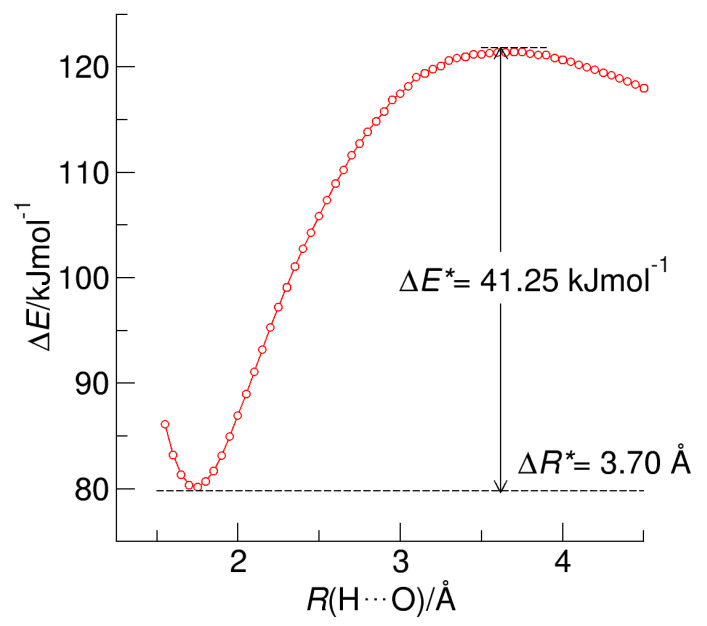
Potential energy curve for the hydrogen-bonded cationic dimer (HOOC–CH_2_–py^+^)_2_, as calculated at the B3LYP–D3/6–31+G* level of theory, relaxing the structure for each hydrogen bond length *R*(H···O). The curve progression is characterized by a clear minimum energy Δ*E* of about 80.17 kJmol^−1^ at the equilibrium bond distance *R*(H···O) = 1.738 Å. The dissociative energy profile shows a pronounced binding well with respect to the dissociated anions and effective equilibrium well depths ΔE* = 41.25 kJmol^−1^ at ΔR* = 3.70 Å with respect to the transition state.

Finally, we address the question of whether complete removal of the anions allows the formation of double-charged (HOOC–(CH_2_)*_n_*–py^+^)_2_ complexes. Here, we focus on the kinetic and thermodynamic stabilities of the (c^+^=c^+^) homodimers. In particular, we want to find out whether there is some perspective to provide first experimental evidence for cationic dimers. The cationic dimer (HOOC–(CH_2_)*_n_*–py^+^)_2_ with *n* = 1 shows a robust local minimum with a clear dissociation barrier preventing “Coulomb explosion” into separated cations. We achieved robust kinetic stabilities while dissociating the dimer along the *R*(H…O) H-bond stretching coordinate with respect to the energy of the isolated cations at Δ*E* = 0. For each intermolecular distance *R*(H…O), all other geometrical variables were optimized at each 0.5 pm step of the scan. The relaxed potential energy curve for the cationic dimer (HOOC–(CH_2_)*_n_*–py^+^)_2_ with *n* = 1 at the B3LYP–D3/6–31+G* level is shown in Figure 4. We indicate the minimum energy Δ*E* = 80.2 kJmol^−1^ at the equilibrium H-bond distance *R*(H…O) = 1.738 Å. The distance *R*(O…O) = 2.737 Å is extremely short too despite the strong repulsive Coulomb interaction present due to only one methylene group spacer between the carboxyl-functionalized groups and the pyridinium rings. The intermolecular oxygen–oxygen distance in this cationic dimer is comparable to that of ice (2.72 Å), exhibiting strong hydrogen bonds, and even 0.14 Å shorter than that of water dimers calculated at the same level of theory [66]. For the dimer (HOOC–CH_2_–py^+^)_2_, we could calculate energy potential curves well beyond the activation barrier height. In Figure 4, we show the effective well depths ΔE* with respect to the transition state at ΔR*(H…O) that signals descent towards separated cations. For this cationic dimer, a distance of about 3.7 Å corresponds to an activation barrier height of about ΔE* = 41.25 kJmol^−1^, showing the enormous strength of the two hydrogen bonds.

### 3.5. Kinetically Stabilized Cationic Dimers (HOOC–(CH_2_)_n_–py^+^)_2_ with n = 1–5

For the dimers (HOOC–(CH_2_)*_n_*–py^+^)_2_ with *n* = 1–5, we show that Δ*E* strongly decreases from 80.2 kJmol^−1^ (*n* = 1) down to –2.5 kJmol^−1^ (*n* = 5), reaching already negative energies. With increasing tether between the carboxylic group and the pyridinium ring of each monomer, a robust kinetic stability switches to energetic stability. Now, the quantum-type short-range attraction wins over classical long-range electrostatic repulsion, providing the first energetically stable cationic dimers. With decreasing Coulomb repulsion, the hydrogen bonds also become more “visible”, indicated by shortened *R*(H…O) and *R*(O…O) H-bond distances (see Table 1).

**Figure 5:**
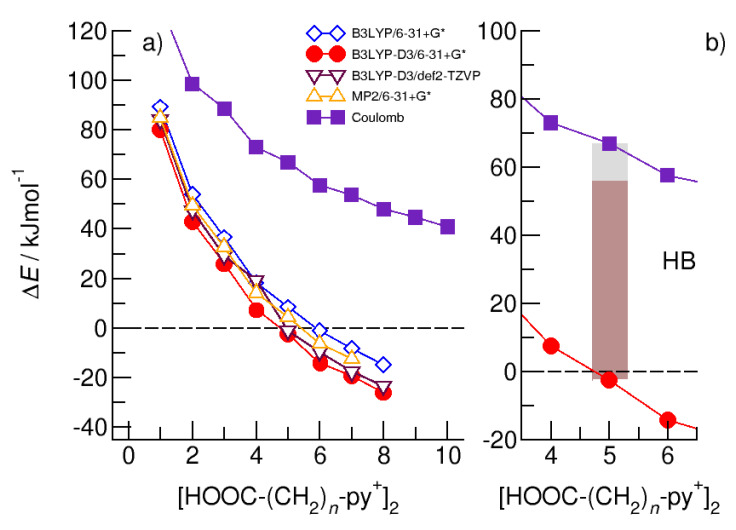
(**a**) The binding energies for the cationic dimers (HOOC−(CH_2_)*_n_*−py^+^)_2_ with *n* = 1–8, as calculated at the dispersion-corrected B3LYP–D3/6–31+G* level of theory (filled circles). For comparison, we added the calculated binding energies obtained at the B3LYP/6−31+G* (diamonds), the B3LYP–D3/def2–TZVP (triangles down), and the MP2/6–31+G* (triangles) levels. The filled squares show the long-range *e*^2^/*R* behavior of idealized Coulomb electrostatic repulsion assuming positive charges *q*= +1 on the centers of the two pyridinium rings as obtained by Mulliken population analysis. (**b**) For the cationic dimer (HOOC–(CH_2_)_5_–py^+^)_2_, the Coulomb repulsion is more than counterbalanced by the attractive hydrogen bond and dispersion forces, resulting in slightly negative Δ*E*= −2.51 kJmol^−1^ (filled circles). The energy difference represents the sum of the H-bond (Δ*E*= −58.5 kJmol^−1^) and dispersion energy (Δ*E*= −10.9 kJmol^−1^), as indicated by the brown and grey bars, respectively.

At *n* = 5, hydrogen bonding and dispersion forces fully compensate for the repulsive Coulomb forces between the cations, allowing for the quantification of the two hydrogen bonds and dispersion interaction. For (HOOC–(CH_2_)*_n_*–py^+^)_2_ with *n* ≥ 5, the binding energies become significantly negative, maximized for (HOOC–(CH_2_)_8_–py^+^)_2_ with the binding energy dropping down to −26.0 kJmol^−1^, as shown in Figure 5. The Mulliken population analysis suggests that a full positive charge (*q* = +1*e*) is located on the pyridinium ring of each cation [65]. Placing +1.0*e* charges at the ring centers and using the DFT-calculated center distances, we can estimate the Coulomb energies for all dimers (HOOC–(CH_2_)*_n_*–py^+^)_2_ with *n* = 1–8. They are shown in comparison with the calculated DFT energies in Figure 5. Because we plot the energies versus the number of methylene groups *n* = 1–8, we observe odd/even effects, which are obviously related to the odd/even changes in the Coulomb repulsion due to changing distances. For the cationic dimer (HOOC–(CH_2_)_5_–py^+^)_2_, the energy release is almost zero (Δ*E*= −2.5 kJmol^−1^). At a distance of about 20.8 Å between the positively charged pyridinium rings, the energy of the cationic dimer is almost the same as that of two isolated cations. For this dimer, the repulsive Coulomb forces are fully counterbalanced by the attractive OH…O hydrogen bonds and dispersion forces that are both described reasonably well by the B3LYP–D3 method. For dissecting an amount of stabilizing energy of about 69.4 kJmol^−1^ into hydrogen bonding and dispersion interaction, we performed additional B3LYP/6–31+G* calculations without including dispersion interaction using Grimme’s D3 method [66,67,68]. Overall, the calculated dispersion energy stabilizes the cation–cation interaction by 10.9 kJmol^−1^. Now we are able to dissect an overall attractive interaction energy of about 69.4 kJmol^−1^. An energy difference of about 58.5 kJmol^−1^ can be related to the two symmetric hydrogen bonds in the cationic dimer. Similar binding energies were reported for formic acid, the archetype of double hydrogen bridges. Kalescky et al. calculated 65.1 kJmol^−1^ from high-level ab initio methods, and Kollipost et al. measured 59.5 kJmol^−1^ from analysis of FTIR spectra recorded in a supersonic slit jet expansion [76,77,78].

**Figure 6:**
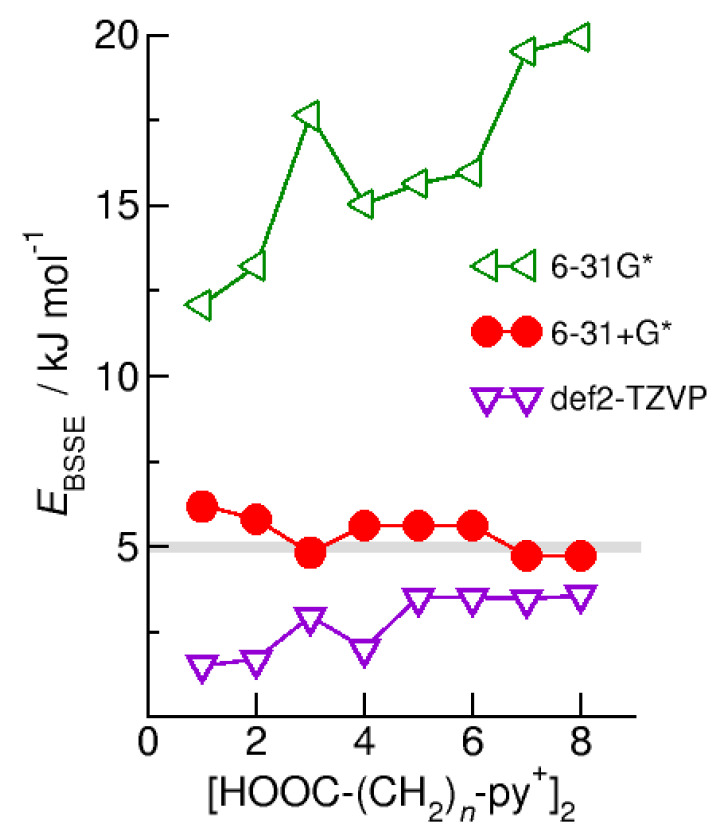
Basis set superposition error (BSSE) calculated for the cationic dimers (HOOC−(CH_2_)*_n_*−py^+^)_2_ with *n* = 1–8 obtained at the B3LYP–D3 level of theory and 6–31G*, 6–31+G*, and def–TZVP basis sets, respectively. Obviously, the counterpoise (CP) approach used here is unreliable for correcting the BSSE in charged systems as reported in the literature [69]. Nevertheless, our calculations show that small Pople basis sets including diffuse function (here 6–31+G*) are well balanced, reducing the BSSE to less than 10% of the overall H-bond energy (Δ*E*= −58.5 kJmol^−1^), which is known for molecular systems [77,78].

As shown in Figure 5, we additionally calculated all cationic dimers at the B3LYP/6–31+G*, the dispersion-corrected B3LYP–D3/def2–TZVP, and the ab initio Møller Plesset MP2/6–31+G* levels of theory for demonstrating the relevance of method and basis set uncertainties [69,70]. Overall, we can state that the influence of the used methods and basis sets is not larger than the effect of adding or removing one methylene group (±3 kJmol^−1^). We point out that the counterpoise (CP) correction of Boys and Bernardi has been questioned recently [71,72]. However, we applied a CP correction to the calculated dimers for demonstrating the minor role of the BSSE correction for the resulting structures and energies if well-balanced basis sets are used (see Figure 6).

### 3.6. Thermodynamically Stabilized Cationic Dimers (HOOC–(CH_2_)_n_–py^+^)_2_ with n = 6–8

We clearly showed that the cationic dimers (HOOC–(CH_2_)*_n_*–py^+^)_2_ with more than five methylene groups exhibit negative binding energies Δ*E*. For *n* = 6–8, we calculated Δ*E* = −14.3 kJmol^−1^, Δ*E* = −19.3 kJmol^−1^, and Δ*E* = −26.0 kJmol^−1^, respectively. The double hydrogen bonds between the two carboxyl-functionalized cations with Δ*E*_HB_ = 58.5 kJmol^−1^ supported by attractive dispersion interaction of about Δ*E*_disp_ = 10.9 kJmol^−1^ in total become stronger than the repulsive Coulomb interaction between them (69.4 vs. 66.8 kJmol^−1^). For judging whether the first cationic dimers with charge *q* = +2 are detectable in gas phase experiments, we have to consider the free energies of the dimeric species as well as their temperature and pressure dependencies. Thus, we calculated the free energies Δ*G*° of all dimers (HOOC–(CH_2_)*_n_*–py^+^)_2_ with a particular focus on (HOOC–(CH_2_)_6_–py^+^)_2_, (HOOC–(CH_2_)_7_–py^+^)_2_, and (HOOC–(CH_2_)_8_–py^+^)_2_ at atmospheric pressure *p* as a function of temperature. For that purpose, the initial frequency calculation for each optimized complex is followed by a thermochemical analysis for varying temperatures and pressures. The equations used for computing thermodynamic data in Gaussian 09 are equivalent to those given in standard statistical texts, such as *Statistical Mechanics* by McQuarrie [79]. The analysis uses the standard expressions for an ideal gas in the canonical ensemble.

**Figure 7:**
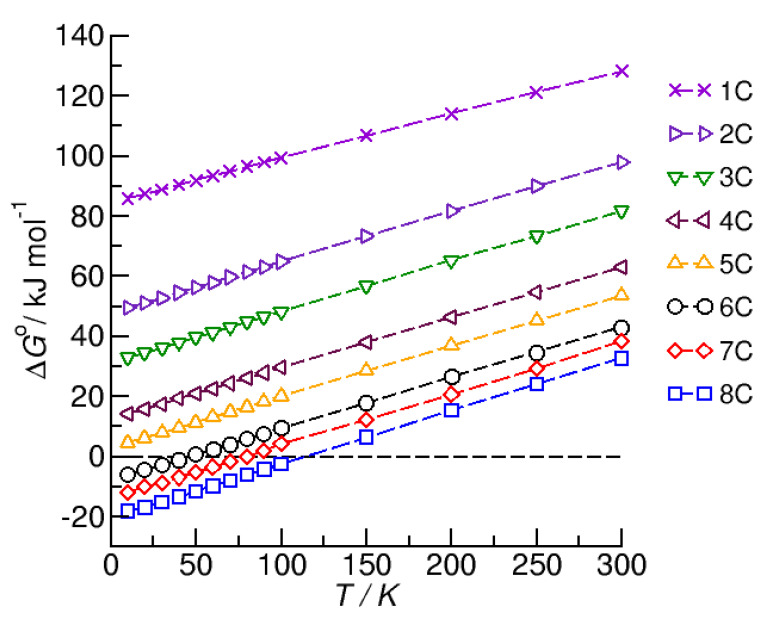
Calculated standard-state free energies Δ*G*° of the cationic dimers (HCOO−(CH_2_)*_n_*−py^+^)_2_ for *n* = 1–8. Negative free energies were calculated for (HCOO−(CH_2_)_6_−py^+^)_2_, (HCOO−(CH_2_)_7_−py^+^)_2_, and (HCOO−(CH_2_)_8_−py^+^)_2_ at temperatures below 47, 80, and 114 K.

**Figure 8:**
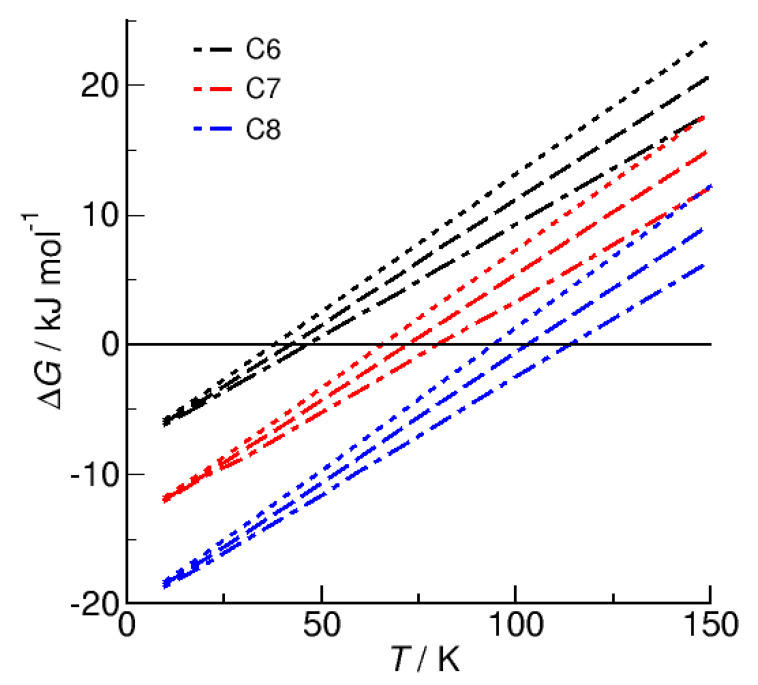
Calculated free energies Δ*G* for cationic dimers (HCOO−(CH_2_)_6_−py^+^)_2_, (HCOO− (CH_2_)_7_−py^+^)_2_, and (HCOO−(CH_2_)_8_−py^+^)_2_. Decreasing pressures in the order of 1.0 (dash-dotted), 0.1 (dashed), and 0.01 (dotted) atm shift the thermodynamic stability to slightly lower temperatures.

In Figure 7, we show that the free energies of all cationic dimers *n* = 1–8 are strongly positive from +135 kJmol^−1^ for *n* = 1 down to +35 kJmol^−1^ for *n* = 8 at 300 K. However, with decreasing temperature, these species can become thermodynamically stable. We obtained Δ*G* = 0 for the dimers (HOOC–(CH_2_)_6_–py^+^)_2_, (HOOC–(CH_2_)_7_–py^+^)_2_, and (HOOC–(CH_2_)_8_–py^+^)_2_ at 47, 80, and 114 K, respectively. Below these temperatures, the cationic dimers win over the monomeric species and should be detectable experimentally. Of course, the pressure dependence is also relevant for the occurrence of these cationic dimers in the gas phase. Thus, we calculated the temperature-dependent free energies at pressures *p* = 1 atm, *p* = 0.1 atm, and *p* = 0.01 atm, respectively. The *p*-dependent free energies in Figure 8 show that the pressure strongly matters at room temperature but is moderate in the low-temperature regime. The thermodynamical stability is shifted down to 38, 66, and 95 K for species *n* = 6–8. However, these dimers are still thermodynamically stable at temperatures where gas phase spectroscopy is usually performed. Thus, the cationic dimers should be detectable in cryogenic ion vibrational predissociation (CIVP) spectroscopy at temperatures below 30 K even at lower pressure [36,37,38,39,40,41,42,43].

### 3.7. QCE Cluster Equilibria

The quantum cluster equilibrium (QCE) of H-bonded liquids introduced by F. Weinhold is based on the role of molecular clusters as fundamental constituent units, characterizable by modern quantum chemistry [50,51,52]. Standard quantum statistical thermodynamic methods are used to treat the equilibria between clusters in the canonical ensemble, leading to predictions of macroscopic thermodynamic properties. The QCE approach suggests close analogies between the covalent interactions of chemical equilibria and the noncovalent interactions of cluster equilibria, enabling one to extend standard techniques of statistical thermodynamics to the weak interaction characteristic of liquefaction and solvation. Meanwhile, a plethora of H-bonded liquids are addressed by the QCE treatment, such as water, ammonia, alcohols, and amides [80,81,82,83,84,85,86,87,88,89,90,91,92,93,94,95,96,97,98,99]. Moreover, quantum cluster equilibrium calculations were extended to binary mixtures, considering dispersion interaction and anharmonic frequencies for the included cluster species [100,101,102,103,104,105,106,107,108]. Other improvements were dedicated to increasing the quality of the partition functions for getting more reliable thermodynamic properties [101]. More recently, QCE has been even used for successfully predicting the ionic product of water [73].

**Figure 9:**
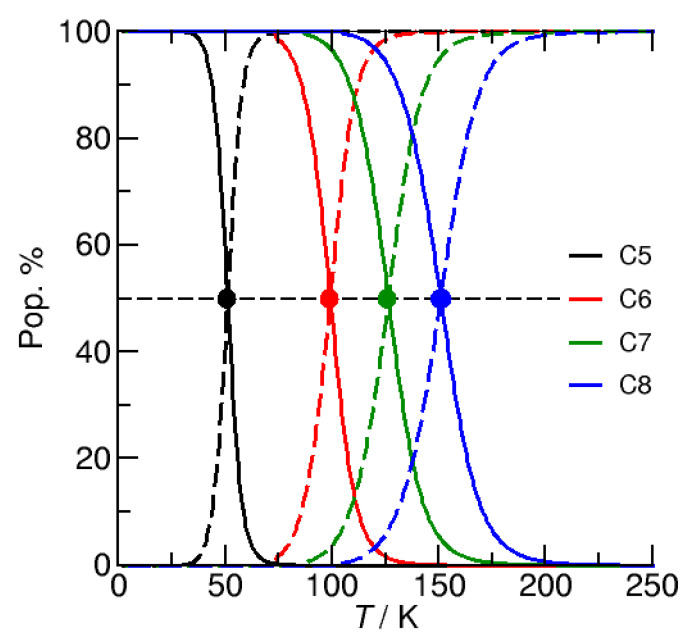
QCE-calculated cluster populations for cationic monomers (dashed lines) and dimers (straight lines) for species *n* = 5–8. Compared with the simple ideal gas approach, we now consider attractive interaction between the clusters’ species expressed by the mean-field parameter *a*_mf_. The filled circles indicate where the species are in the equilibrium (Δ*G* = 0 kJmol^−1^). Here, we show the data for varying values *a*_mf_ (from 0.04 to 0.1), which is equivalent to assuming the dispersion interaction between the species ranging from 1.2 to 3 kJmol^−1^, shifting the equilibrium to about 40 K higher temperatures.

Here, we used the QCE approach as implemented in the Peacemaker software package [55]. Usually, two standard-state reference values are employed to determine the two parameters *a*_mf_ and *b*_xv_, such that the resulting isobar reproduces: (a) the experimental density of liquids at 298.15 K and (b) their boiling points. In this work, we used QCE calculations for deriving the gas phase equilibria between pure cationic dimers and their monomeric units. For that purpose, we used a fixed value, *b*_xv_ = 1, but varied the *a*_mf_ parameter for taking intermolecular forces between the species into account. This mean-field approach allows for studying the cluster equilibria as a function of the temperature and pressure. In Figure 9, we show the cluster populations of the cationic monomers and dimers for species *n* = 5–8. We used values between 0.04 and 0.1 for the mean-field parameter *a*_mf_, which is equivalent to introducing intermolecular interaction energies of between 1.2 and 3 kJmol^−1^, respectively. If such a moderate intermolecular interaction is assumed, the 50% populations of cationic dimers are already achieved at 51, 99, 126, and 151 K for *n* = 5–8. Thus, the experimental situation for detecting the pure cationic dimers is even more comfortable, if the dispersion interaction between the gas phase species is considered.

### 3.8. NBO Descriptors Strongly Correlate with Energy, H-Bond Distances, NMR Proton Chemical Shifts, and Vibrational Frequencies

**Figure 10:**
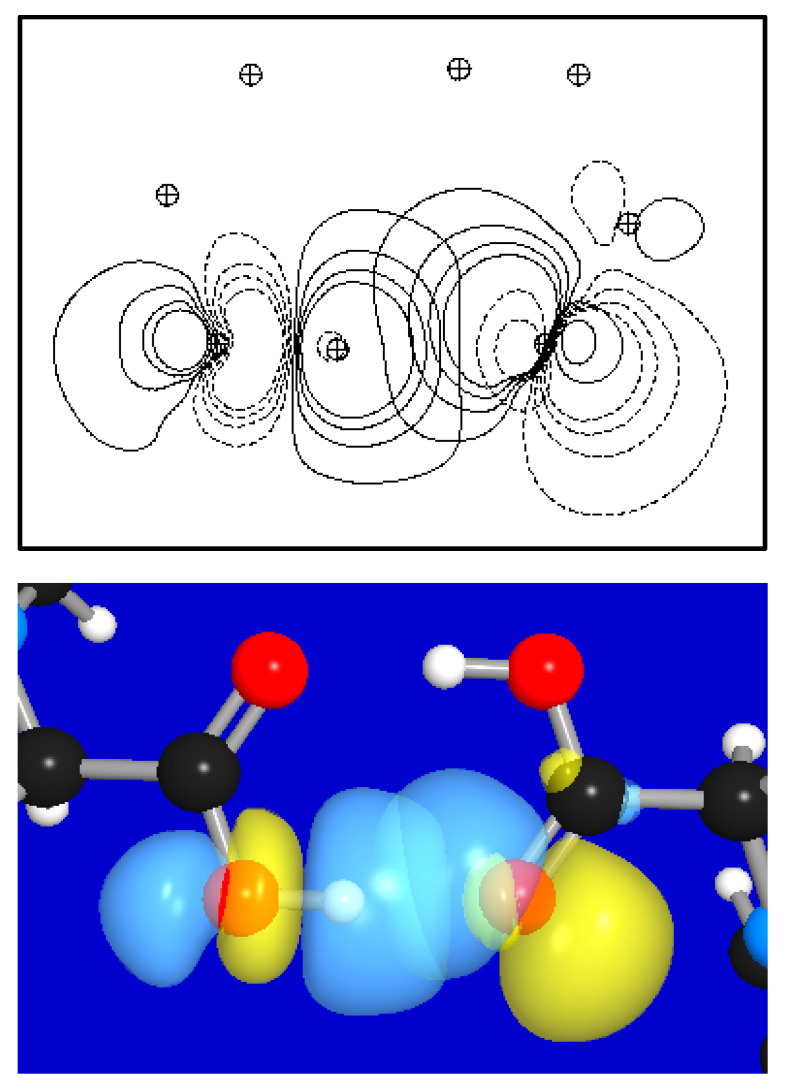

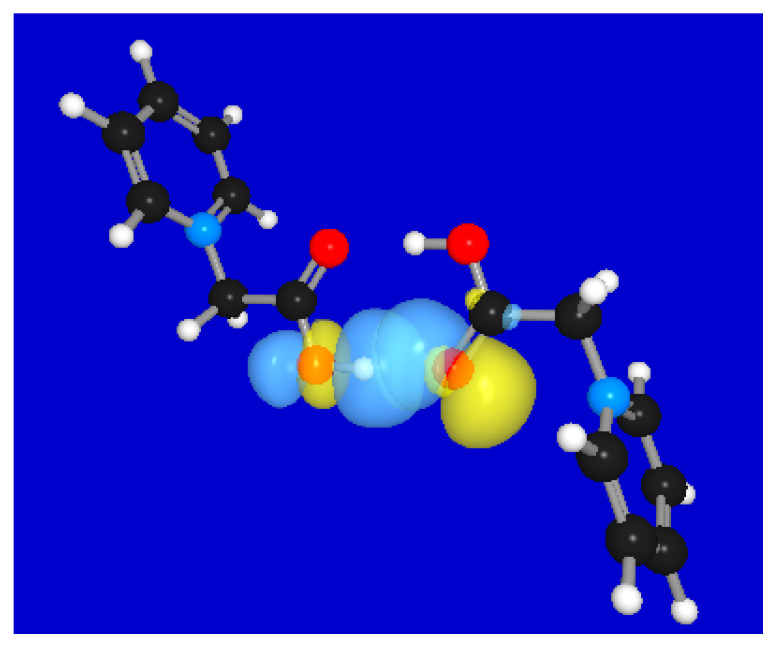
NBO 2D contour and 3D overlap diagrams for n_O_ → σ*_OH_ donor–acceptor interaction in the cationic dimer (HCOO–CH_2_–py^+^)_2_ equilibrium geometry with estimated second-order Δ*E*^(2)^_n→σ*_ = 27.5 kcal·mol^−1^ interaction.

The cationic dimers demonstrate that the short-range donor–acceptor covalency forces successfully compete with the powerful long-range electrostatic repulsions [22,23,24,25,26,27,28,29,30,31,32,33,34,35]. The characteristic covalency features of the double (H…O) hydrogen bonds can be readily recognized in the framework of the natural bond orbital (NBO) analysis as distinctive n*_O_* → σ*_OH_ donor–acceptor interactions expressed by the second-order stabilization energies Δ*E*^(2)^_n→σ*_ and estimated total charge transfers *q*_CT_ (see Figure 10) [56,57,58,59,60]. The charge from the oxygen lone pair orbitals of the carboxyl group CO of one cation is donated into the OH antibond orbital of the hydroxyl group of the second anion and vice versa. This way, the short-range donor–acceptor covalency forces overcome the strong long-range electrostatic repulsive forces as expected for ions of like charge. Here, we show that the NBO approach is also suitable for describing the energetic and spectroscopic feature of hydrogen-bonded ions of like charge.

**Figure 11:**
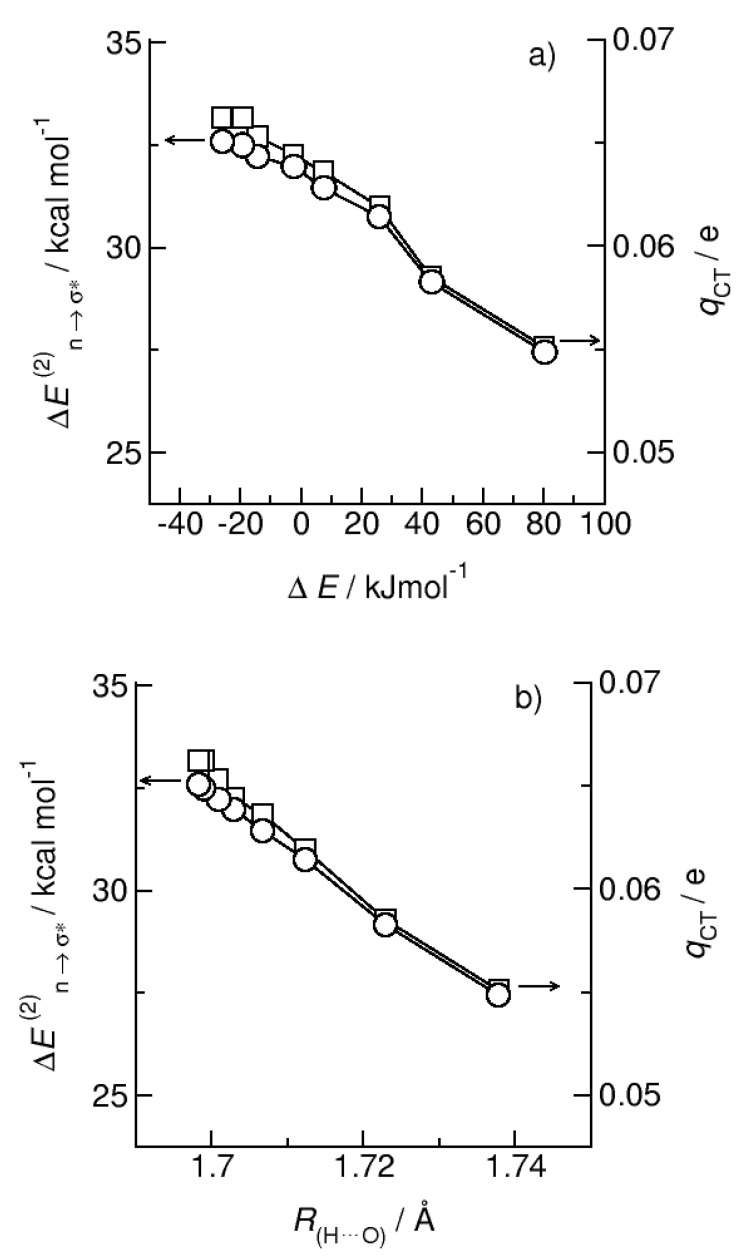

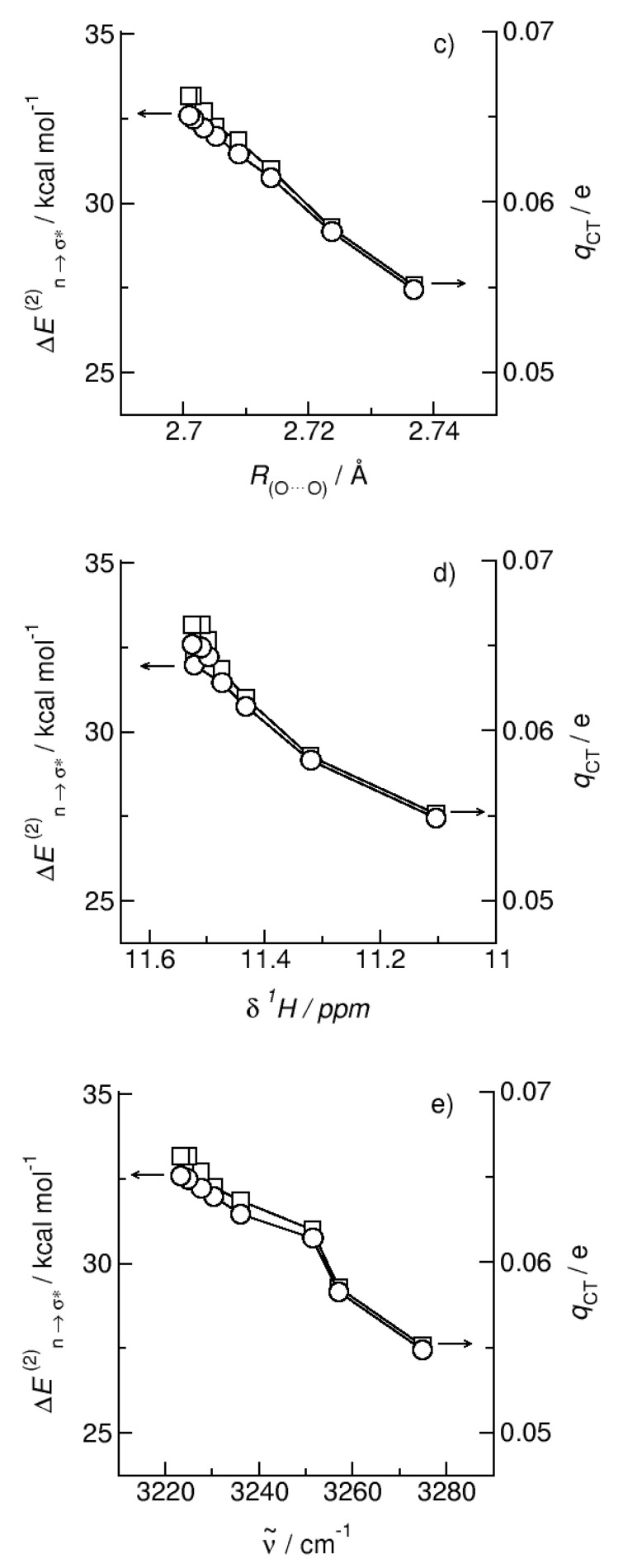
NBO-calculated second-order stabilization energies Δ*E*(2)_n→σ*_ (open circles) and estimated total charge transfers *q*_CT_ (closed squares) for cationic dimers *n* = 1–8 plotted versus calculated (**a**) energies Δ*E*, (**b**) intermolecular hydrogen bond distances, *R*(H···O), (**c**) intermolecular hydrogen bond *R*(O···O), (**d**) NMR proton chemical shifts, δ*^1^*H (relative to the standard tetramethylsilane, TMS), and (**e**) OH vibrational frequencies $\tilde{\nu }$(OH). The almost linear dependences showing slight odd/even effects indicate the strong relation between NBO stabilization energies and charge transfers with energetic, geometric, and spectroscopic properties of the cationic dimers.

In Figure 11a–e, we show the NBO energy descriptors versus binding energies Δ*E*, H-bond distances *R*(H…O) and *R*(O…O), and proton chemical shifts of the hydroxyl protons δ^1^H (vs. TMS) and infrared vibrational frequencies, $\tilde{\nu }$(OH), for all dimers *n* = 1–8. As shown, the second-order stabilization energies Δ*E*^(2)^_n→σ*_ and the total charge transfers *q*_CT_ are strongly correlated with the enhanced H-bond strength in the order of *n* = 1 to *n* = 8 governed by the decreasing Coulomb repulsion with an increasing number of methylene groups between the functional groups and the pyridinium rings. Obviously, this effect is almost saturated for species *n* = 6–8, wherein attractive hydrogen bonding and dispersion interaction are larger than the repulsive Coulomb interaction. This behavior is reflected in lower energies Δ*E*, shorter H-bond distances *R*(H…O) and *R*(O…O), enhanced downfield chemical shifts δ^1^H, and redshifted vibrational frequencies *ν*_OH_. A change in *R*(H…O) and *R*(O…O) distances of about 0.4 Å between dimers *n* = 1 and *n* = 8 is correlated with downfield chemical shifts δ^1^H of about 0.45 ppm and redshifted vibrational frequencies$\;\tilde{\nu }$(OH) of about 55 cm^−1^. This demonstrates the usefulness of NBO analysis and highlights NMR and IR spectroscopies as the most sensitive probes of hydrogen bonding, also for characterizing the interaction strengths between ions of like charge. Overall, the NBO parameters strongly indicate the covalent and anti-electrostatic character of hydrogen bonding in cationic dimers.

## 4. Conclusions

We show that carboxyl-functionalized ionic liquids (ILs) form doubly hydrogen-bonded cationic dimers (c^+^=c^+^) despite the repulsive forces between ions of like charge and competing hydrogen bonds between cation and anion (c^+^–a^−^). After detecting the structural motif of doubly hydrogen-bonded cationic dimers (c^+^=c^+^) in the solid state of the ionic liquid 1–(carboxymethyl)pyridinium bis(trifluoromethylsulfonyl)imide [HOOC–CH_2_–py][NTf_2_] by means of X-ray diffraction, we explored differently hydrogen-bonded isomers of neutral (HOOC–(CH_2_)*_n_*–py^+^)_2_(NTf_2_^−^)_2_, single-charged (HOOC–(CH_2_)*_n_*–py^+^)_2_(NTf_2_^−^), and double-charged (HOOC–(CH_2_)*_n_*–py^+^)_2_ complexes for demonstrating the paradoxical case of “anti-electrostatic” hydrogen bonding (AEHB) between ions of like charge. In the neutral complex (HOOC–CH_2_–py^+^)_2_(NTf_2_^−^)_2_, the double hydrogen bonds in the (c^+^=c^+^)(a^−^)^2^ complex are stronger than the two hydrogen bonds in a (c^+^–c^+^–a^−^)(a^−^) ^2^ complex and the two isolated hydrogen bonds in a (c^+^–a^−^)^2^ complex. Release of one counterion from the neutral system resulting in positively charged (HOOC–CH_2_–py^+^)_2_NTf_2_^–^ complexes shows that the double hydrogen bonds in the (c^+^=c^+^)(a^−^) complex remain more stable than the two hydrogen bonds in each of the (c^+^–c^+^–a^−^) and (c^+^–a^−^)^2^ complexes. Fully removing the anions resulting in doubly hydrogen-bonded cationic dimers (HOOC–(CH_2_)*_n_*–py^+^)_2_ still provides kinetic stability for the (c^+^=c^+^) homodimers. At about *n* = 5, hydrogen bonding and dispersion forces fully compensate for the repulsive Coulomb forces between the cations, allowing for the quantification of the two equivalent hydrogen bonds and dispersion interaction in the order of 58.5 and 11 kJmol^−1^, respectively. For getting even thermodynamic stability for the cationic dimers (HOOC–(CH_2_)*_n_*–py^+^)_2_, we further increased the alkyl chain length for tethering the ions of like charge and reducing the Coulomb repulsion. For *n* ≥ 5, quantum-type short-range attraction wins over classical long-range electrostatic repulsion, resulting in negative binding energies and providing the first energetically stable anionic dimers. For *n* = 6–8, we calculated negative free energies below 47, 80, and 114 K at ambient pressure, respectively. Decreasing the pressure down to 0.01 atm still provides cationic dimers, which should be detectable by means of cryogenic ion vibrational spectroscopy at temperatures below 30 K. QCE theory predicts the equilibria between cationic monomers and dimers by considering intermolecular interaction among the species. We rationalize the H-bond characteristics of the dimers by the natural bond orbital (NBO) approach. NBO-based CT descriptors demonstrate strong correlative relationships with known experimental signatures of hydrogen bonding, such as NMR chemical shifts and IR vibrational frequencies. Overall, we show that the double hydrogen bond motif is already present in the condensed phase of the carboxyl-functionalized IL with *n* = 1, and that there is hope to find the first pure cationic dimers with *n* = 6–8 in gas phase experiments at a low temperature.

## Figures and Tables

**Table 1:** Binding energies Δ*E* and intermolecular distances *R*(H…O) of the optimized cationic dimers (HOOC–(CH_2_)*_n_*–py^+^)_2_ with *n* = 1–8 calculated at the B3LYP–D3/6–31+G* level of theory.

*n*	*R*(H…O)/Å	*R*(O…O)/Å	Δ*E*/kJmol^−1^
1	1.738	2.737	80.1
2	1.723	2.724	42.9
3	1.712	2.714	35.91
4	1.707	2.709	7.4
5	1.703	2.705	−2.5
6	1.701	2.703	−14.3
7	1.670	2.702	−19.3
8	1.698	2.701	−26.0

## Supplementary Materials

The following supporting information can be downloaded, CIF/PLATON report: Datablock: av_hcoomepyntf2.

## Sample Availability

Samples of the compound 1−(carboxymethyl)pyridinium bis(trifluoromethylsulfonyl)imide are available from the authors.
