# Supplementary material for: Thermodynamically Stable Cationic Dimers in Carboxyl-Functionalized Ionic Liquids: The Paradoxical Case of “Anti-Electrostatic” Hydrogen Bonding
Source: Molecules. 2022 Jan 7;27(2):366. doi: 10.3390/molecules27020366 (PMC8778807; doi:10.3390/molecules27020366)

checkCIF/PLATON report

Structure factors have been supplied for datablock(s) av_hcoomepyntf2

THIS REPORT IS FOR GUIDANCE ONLY. IF USED AS PART OF A REVIEW PROCEDURE FOR PUBLICATION, IT SHOULD NOT REPLACE THE EXPERTISE OF AN EXPERIENCED CRYSTALLOGRAPHIC REFEREE.

No syntax errors found.   CIF dictionary   Interpreting this report

Datablock: av_hcoomepyntf2

```
Bond precision:  C-C = 0.0045 A          Wavelength=0.71073

Cell:           a=8.1051(5)       b=8.1374(6)      c=13.7868(11)
                alpha=77.024(3)   beta=77.263(2)   gamma=62.151(2)
Temperature:    123 K
```

	Calculated	Reported
Volume	776.38(10)	776.38(10)
Space group	P 1	P 1
Hall group	P 1	P 1
Moiety formula	C2 F6 N O4 S2, C7 H8 N O2	C7 H8 N O2 +, C2 F6 N O4 S2 -
Sum formula	C9 H8 F6 N2 O6 S2	C9 H8 F6 N2 O6 S2
Mr	418.29	418.29
Dx,g cm-3	1.789	1.789
Z	2	2
Mu (mm-1)	0.442	0.442
F000	420.0	420.0
F000’	420.86	
h,k,lmax	11,11,19	11,11,19
Nref	9882[4941]	9718
Tmin,Tmax	0.885,0.957	0.652,0.747
Tmin’	0.838	

```
Correction method= # Reported T Limits: Tmin=0.652 Tmax=0.747
AbsCorr = MULTI-SCAN

Data completeness= 1.97/0.98      Theta(max)= 30.999

R(reflections)= 0.0328( 8564)     wR2(reflections)= 0.0686( 9718)

S = 1.035                  Npar= 460
```

The following ALERTS were generated. Each ALERT has the format
test-name_ALERT_alert-type_alert-level.
Click on the hyperlinks for more details of the test.

Alert level C

```
PLAT340_ALERT_3_C Low Bond Precision on  C-C Bonds ...............     0.0045 Ang.
PLAT911_ALERT_3_C Missing FCF Refl Between Thmin & STh/L=    0.600          3 Report
```

Alert level G

```
PLAT033_ALERT_4_G Flack x Value Deviates > 3.0 * sigma from Zero .      0.290 Note
PLAT042_ALERT_1_G Calc. and Reported Moiety Formula Strings Differ     Please Check
PLAT242_ALERT_2_G Low    'MainMol' Ueq as Compared to Neighbors of        C17 Check
PLAT242_ALERT_2_G Low    'MainMol' Ueq as Compared to Neighbors of        C18 Check
PLAT432_ALERT_2_G Short Inter X...Y Contact  O5       ..C8              2.90 Ang.
                                                     x,y,z  =      1_555 Check
PLAT432_ALERT_2_G Short Inter X...Y Contact  O7       ..C1              2.94 Ang.
                                                     x,y,z  =      1_555 Check
PLAT432_ALERT_2_G Short Inter X...Y Contact  O8       ..C1              2.95 Ang.
                                                   1+x,y,z  =      1_655 Check
PLAT432_ALERT_2_G Short Inter X...Y Contact  O12      ..C2              2.98 Ang.
                                                     x,y,z  =      1_555 Check
PLAT434_ALERT_2_G Short Inter HL..HL Contact F2       ..F6              2.71 Ang.
                                                  x,-1+y,z  =      1_545 Check
PLAT434_ALERT_2_G Short Inter HL..HL Contact F3       ..F4              2.77 Ang.
                                                1+x,-1+y,z  =      1_645 Check
PLAT434_ALERT_2_G Short Inter HL..HL Contact F8       ..F10             2.82 Ang.
                                                -1+x,1+y,z  =      1_465 Check
PLAT883_ALERT_1_G No Info/Value for _atom_sites_solution_primary .     Please Do !
PLAT910_ALERT_3_G Missing # of FCF Reflection(s) Below Theta(Min).          1 Note
PLAT912_ALERT_4_G Missing # of FCF Reflections Above STh/L=  0.600          2 Note
PLAT913_ALERT_3_G Missing # of Very Strong Reflections in FCF ....          2 Note
PLAT933_ALERT_2_G Number of OMIT Records in Embedded .res File ...          3 Note
PLAT978_ALERT_2_G Number C-C Bonds with Positive Residual Density.          5 Info
```

---

0 **ALERT level A** = Most likely a serious problem - resolve or explain
0 **ALERT level B** = A potentially serious problem, consider carefully
2 **ALERT level C** = Check. Ensure it is not caused by an omission or oversight
17 **ALERT level G** = General information/check it is not something unexpected

2 ALERT type 1 CIF construction/syntax error, inconsistent or missing data
11 ALERT type 2 Indicator that the structure model may be wrong or deficient
4 ALERT type 3 Indicator that the structure quality may be low
2 ALERT type 4 Improvement, methodology, query or suggestion
0 ALERT type 5 Informative message, check

It is advisable to attempt to resolve as many as possible of the alerts in all categories. Often the minor alerts point to easily fixed oversights, errors and omissions in your CIF or refinement strategy, so attention to these fine details can be worthwhile. In order to resolve some of the more serious problems it may be necessary to carry out additional measurements or structure refinements. However, the purpose of your study may justify the reported deviations and the more serious of these should normally be commented upon in the discussion or experimental section of a paper or in the "special_details" fields of the CIF. checkCIF was carefully designed to identify outliers and unusual parameters, but every test has its limitations and alerts that are not important in a particular case may appear. Conversely, the absence of alerts does not guarantee there are no aspects of the results needing attention. It is up to the individual to critically assess their own results and, if necessary, seek expert advice.

Publication of your CIF in IUCr journals

A basic structural check has been run on your CIF. These basic checks will be run on all CIFs submitted for publication in IUCr journals (*Acta Crystallographica*, *Journal of Applied Crystallography*, *Journal of Synchrotron Radiation*); however, if you intend to submit to *Acta Crystallographica Section C* or *E* or *IUCrData*, you should make sure that full publication checks are run on the final version of your CIF prior to submission.

Publication of your CIF in other journals

Please refer to the *Notes for Authors* of the relevant journal for any special instructions relating to CIF submission.

PLATON version of 03/06/2021; check.def file version of 02/06/2021

Datablock av_hcoomepyntf2 - ellipsoid plot

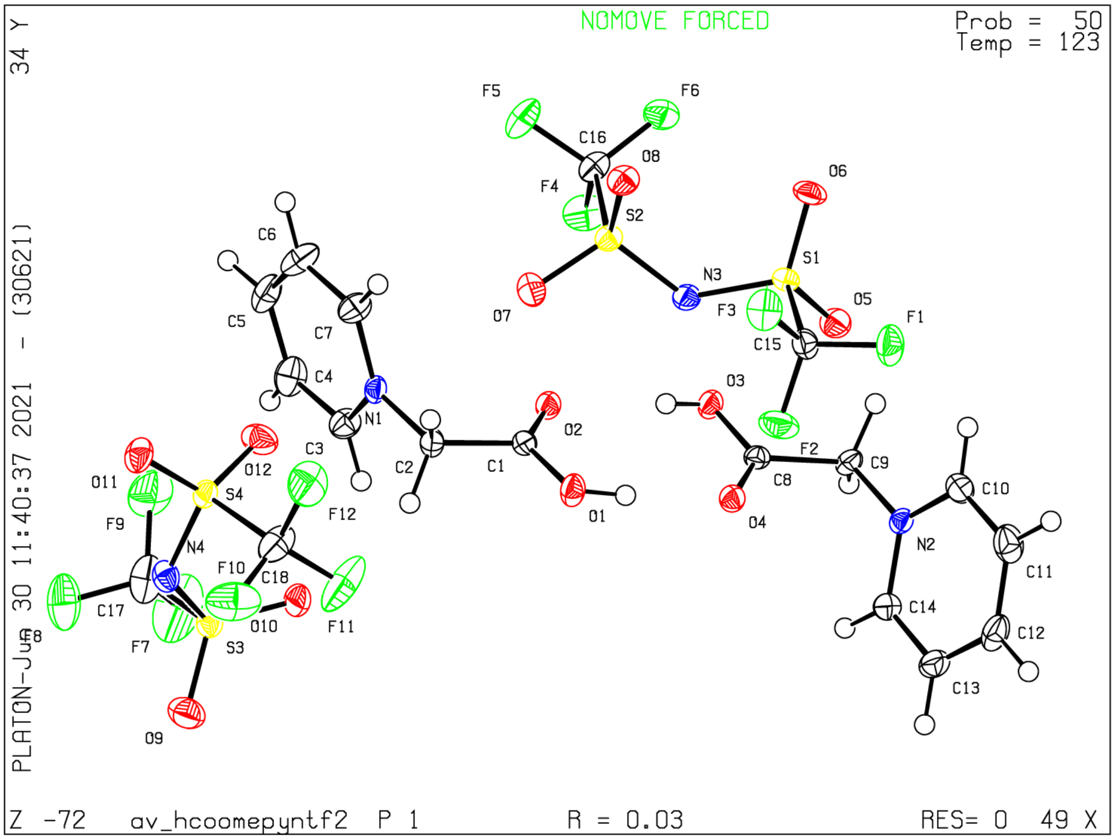

Supplement: Supplementary file 1 [file molecules-27-00366-s001.zip › molecules-1511519-supplementary.pdf]
